# Supplementary material for: Faunal Diet of Adult Cane Toads, Rhinella marina, in the Urban Landscape of Southwest Florida
Source: Animals (Basel). 2023 Sep 13;13(18):2898. doi: 10.3390/ani13182898 (PMC10526063; doi:10.3390/ani13182898)
Supplement: Supplementary file 1 [file animals-13-02898-s001.zip › Table S1. Cane toad diet studies.pdf]

Table S1. Summary of cane toad diet studies in native and introduced ranges. For Diet metric, an index of relative importance (IRI; [51]) was calculated if number, frequency, and volume/mass were available, and an alimentary importance index (AII; [49]) was calculated if only two of the component metrics were available.

Range	Region	Location/study	N	Habitat	Life stage	Diet metric	Primary	Secondary	Tertiary	Comments
Native	Central America	Rio Grande, Nicaragua<br>Noble, 1918 [96]	"few"	Urban	n/a	Frequency	Blattodea (large cockroaches)<br>n/a			Unquantified comment from text.
Native	Central America	Cocole, Panama<br>Zug and Zug, 1979 [8]	9	Grassy gravel pit	Juvenile - adult	Frequency	Formicidae (ants)<br>100%	Carabidae (ground beetles)<br>67%	Coleoptera ("oblong" beetles)<br>56%	% frequency calculated from Table C in Appendix 1; oblong beetles include Elateridae (click beetles)/Cermabycidae (longhorn beetles)/Lampyridae (fireflies).
Native	Central America	Gamboa, Panama<br>Zug and Zug, 1979 [8]	10	Urban	Juvenile - adult	Frequency	Formicidae (ants)<br>80%	Carabidae (ground beetles)<br>70%	Diplopoda (millipedes) and Gastropoda (snails)<br>50%	% frequency calculated from Table C, Appendix 1.
Native	Central America	Los Santos, Panama<br>Zug and Zug, 1979 [8]	12	Scrub and patchy grass	Juvenile - adult	Frequency	Formicidae (ants)<br>83%	Scarabaeidae (scarab beetles) and Coleoptera ("oblong" beetles)<br>25%	Winged Hymenopterans (bees and wasps)<br>17%	% frequency calculated from Table C, Appendix 1; oblong beetles include Elateridae (click beetles)/Cermabycidae (longhorn beetles)/Lampyridae (fireflies).
Native	Central America	Summit Hill, Panama<br>Zug and Zug, 1979 [8]	11	Golf course	Juvenile - adult	Frequency	Scarabaeidae (scarab beetles)<br>82%	Winged Hymenoptera (bees and wasps) and Coleoptera ("oblong" beetles)<br>73%	Formicidae (ants)<br>55%	% frequency calculated from Table C, Appendix 1; oblong beetles include Elateridae (click beetles)/Cermabycidae (longhorn beetles)/Lampyridae (fireflies).
Native	South America	Trinidad & British Guiana<br>Weber, 1938 [97]	28	Urban/clearings	n/a	Frequency	*Ectatomma ruidum* (Neotropical ant)<br>57%	Coleoptera (beetle fragments and larvae)<br>39%	*Pheidole fallax* (big-headed ant)<br>36%	% frequency calculated from the list of stomach contents and assuming 28 samples as described in the text.
Native	South America	Santa Cecilia, Ecuador<br>Duellman, 1978 [98]	25	River banks and clearings	n/a	Frequency	Formicidae (large ants)<br>68%	Coleoptera (beetles)<br>64%	Orthoptera (crickets and grasshoppers)<br>40%	% frequency from the text.
Native	South America	Brazil, Rio Tapajós<br>Strüssmann et al., 1984 [82]	29	Sandy river banks	Adult	AII	Hymenoptera (ants)<br>56%	Isoptera/Blattodea (termites)<br>39%	Coleoptera (beetles)<br>4%	% number and % frequency from combined Fig. 3F and 3H used to calculate % AII.
Native	South America	Golfito National Wildlife Refuge, Costa Rica<br>Cabrera et al., 1996 [99]	55	n/a	Adult	Frequency	Hymenoptera (ants)<br>58%	Coleoptera (beetles)<br>17%	Myriapoda (millipedes and centipedes)<br>17%	% frequency estimated from two larger size classes (>77 mm) from Fig. 1.; no signif. diff. by sex.
Native	South America	Venezuela<br>Evans and Lampo, 1996 [29]	201	Combined habitats	Adult	Mass	Coleoptera (beetles)<br>27%	Formicidae (ants)<br>13%	Odonata (larval dragonflies and damselflies)<br>8%	% mean mass from the text.
Native	South America	Venezuela<br>Evans and Lampo, 1996 [29]	61	Arid	Adult	Mass	Coleoptera (beetles)<br>28%	Odonata (larval dragonflies and damselflies)<br>21%	Formicidae (ants)<br>12%	% mean mass from Table 2; seasonal diff.
Native	South America	Venezuela<br>Evans and Lampo, 1996 [29]	42	Savanna	Adult	Mass	Coleoptera (beetles)<br>55%	Orthoptera (grasshoppers and crickets) and Formicidae (ants)<br>3%	Odonata (larval dragonflies and damselflies)<br>1%	% mean mass from Table 2; seasonal diff.
Native	South America	Venezuela<br>Evans and Lampo, 1996 [29]	47	Forest/scrub	Adult	Mass	Formicidae (ants)<br>28%	Orthoptera (grasshoppers and crickets) and Coleoptera (beetles)<br>9%	Odonata (larval dragonflies and damselflies)<br>5%	% mean mass from Table 2; seasonal diff.
Native	South America	Cuzco Amazonico, Peru<br>Parmelee, 1999 [9]	5	Forest clearing	Adult	IRI	Formicidae (ants)<br>61%	Coleoptera (beetles)<br>20%	Pentatomidae (stink bugs),Hemiptera (true bugs), and Isoptera (termites)<br>6%	% frequency calculated and % number and % volume in Appendix 2 were used to calculate IRI.
Native	South America	Colombia<br>Isaacs and Hoyos, 2010 [100]	49	Coffee farms	n/a	Frequency	Coleoptera (beetles, including Curculonidae and Elateridae)<br>61%	Hymenoptera (ants, including *Atta*, *Pheidole*, and *Odontomachus*)<br>27%	Araneae (spiders)<br>18%	% frequency calculated from Table 1 using totals for lower resolution taxa; no signif. diff. by sex.
Native	South America	Sucre, Colombia<br>Sampedro-Marín et al., 2011 [87]	approx. 87	Forest/savannah - dry	Adult	AII	Hymenoptera (ants)<br>91%	Coleoptera (beetles)<br>7%	Arachnids (spiders)<br>1%	Reported importance index is a partial calculation of AII; reported seasonal values from Table 3 were summed and used to calculate % AII; no signif. diff. by season or sex.
Native	South America	Sucre, Colombia<br>Sampedro-Marín et al., 2011 [87]	approx. 111	Forest/savannah - wet	Adult	AII	Hymenoptera (ants)<br>78%	Coleoptera (beetles)<br>21%	Diplopoda (millipedes)<br>1%	Reported importance index is a partial calculation of AII; reported seasonal values from Table 3 were summed and used to calculate % AII; no signif. diff. by season or sex.
Native	South America	Colombia<br>Blanco-Torres et al., 2020 [101]	44	Tropical dry forests	Subadult - adult	Number	*Rhynchotermes* (nasute termites)<br>41%	*Labidus* (army ants)<br>13%	*Pheidole* (big-headed ants)<br>8%	% number calculated from supplementary digital database; numerous other species were identified in the top percent such as *Atta* and *Solenopsis*.
Introduced	Carribean	Puerto Rico<br>Dexter, 1932 [26]	301	Agricultural	n/a	AII	*Phyllophaga* sp. (May beetles)<br>43%	Diplopoda (millipedes)<br>35%	*Diaprepes spengleri*? (root weevil)<br>15%	% number and % "bulk" from Table XI used to calculate % AII.
Introduced	Carribean	Puerto Rico<br>Wolcott, 1937 [27]	89 pellets	Urban	n/a	Number	*Dyscinetus barbatus* (rice beetles)<br>51%	*Phyllophaga* spp. (May beetles)<br>44%	*Diaprepes abbreviatus* (root weevil)<br>2%	% number calculated for fecal pellets from Rio Piedras in Table 1.
Introduced	North America	Southeast Florida<br>Krakauer, 1968 [16]	85	Urban	Adult	Frequency	Coleoptera (beetles)<br>61%	Formicidae (ants)<br>28%	Dermaptera (earwigs) and Gastropoda (snails)<br>21%	% frequency from Table 2.
Introduced	North America	West-central Florida<br>Rossi, 1981 [20]	34	Urban	Adult	Frequency	Coleoptera (beetles)<br>44%	Formicidae (ants)<br>35%	Blattidae (cockroaches)<br>15%	% frequency from Table 2; mammal hair had a high frequency (21%) but was probably scavenged.
Introduced	North America	Central Florida<br>Meshaka and Powell, 2010 [33]	95	Urban	Subadult - adult	Importance	Coleoptera (beetles)<br>70%	Isoptera/Blattodea (termites)<br>38%	Hymenoptera (ants)<br>37%	% importance inferred from Fig. 3; the Importance Value was referenced as %N+%V+%F but these diet metrics were not provided.
Introduced	North America	Southwest Florida<br>Current study	239	Golf course communities	Adult	IRI	*Pheidole* sp. (big-headed ants)<br>35%	*Anadenobolus monilicornis* (yellow-banded millipedes)<br>27%	*Sphenophorus venatus* (hunting billbug)<br>19%	
Introduced	North America	Southwest Florida<br>Current study	120	Golf course community A	Adult	IRI	*Sphenophorus venatus* (hunting billbug)<br>42%	*Pheidole* sp. (big-headed ants)<br>23%	*Anadenobolus monilicornis* (yellow-banded millipedes)<br>15%	
Introduced	North America	Southwest Florida<br>Current study	119	Golf course community B	Adult	IRI	*Anadenobolus monilicornis* (yellow-banded millipedes)<br>41%	*Pheidole* sp. (big-headed ants)<br>38%	Lepidoptera (moths and butterflies), primarily larvae<br>8%	
Introduced	North Atlantic	Bermuda<br>Linzey et al., 1998 [30]	31	Urban/marsh - cool season	Adult	AII	Diplopoda (millipedes)<br>51%	Lepidoptera larvae (moths and butterflies) and Curculionidae (weevils)<br>11%	Formicidae (ants)<br>8%	% frequency and % volume for fauna (no vegetation) from Table 2 used to calculate % AII; seasonal diff.
Introduced	North Atlantic	Bermuda<br>Linzey et al., 1998 [30]	44	Urban/marsh - hot season	Adult	AII	Diplopoda (millipedes)<br>62%	Orthoptera/Blattidae (cockroaches)<br>23%	Isopoda (sowbugs)<br>4%	% frequency and % volume for fauna (no vegetation) from Table 2 used to calculate % AII; seasonal diff.
Introduced	West Pacific	Fiji<br>Hinckley, 1963 [102]	100	Agricultural/urban	Juvenile - adult	Number	Formicidae (ants, including *Odontomachus*, *Tapinoma*, and *Pheidole*)<br>34%	Lepidoptera larvae (moths, primarily *Pseudaletia separata*)<br>16%	Diplipoda (millipedes, including *Asiomorpha* and *Trigoniulus*)<br>13%	% number calculated from Table I, using totals for lower resolution taxa.
Introduced	West Pacific	Papua New Guinea<br>Zug et al., 1975 [28]	476	Savanna road	Subadult - adult	Number	Isoptera/Blattodea (termites)<br>39%	Formicidae (ants, including *Polyrhachis*, *Pheidole*, and *Odontomachus*)<br>33%	Lepidoptera (moths and butterflies)<br>8%	% number calculated from Appendix 2, using totals for lower resolution taxa.
Introduced	West Pacific	Papua New Guinea<br>Zug et al., 1975 [28]	37	Rainforest road	Subadult - adult	Number	Formicidae (ants, including *Odontomachus* and *Pheidole*)<br>61%	Curculionidae (weevils)<br>8%	Scarabaeidae (scarab beetles)<br>6%	% number calculated from Appendix 3, using totals for lower resolution taxa: Lepidoptera and Diplopoda close quarternary (5%) prey.
Introduced	West Pacific	Papua New Guinea<br>Bailey, 1976 [103]	162	Cacao plantation	n/a	Frequency	*Subulina octina* (terrestrial snail)<br>37%	*Odontomachus simillimus* (trap-jaw ant)<br>25%	Oecophylla smaragdina (Asian weaver ant)<br>15%	% frequency based on text in the results section.
Introduced	West Pacific	Northeastern Queensland, Australia<br>Werren and Trenerry, 1993 [81]	257 total	Upland rainforest	Adult	Frequency	Coleoptera (beetles, excluding weevils)<br>86%	Formicidae (ants, excluding bull ants and green tree ants)<br>79%	Curculionidae (weevils)<br>53%	% frequency from Table 1.
Introduced	West Pacific	Northeastern Queensland, Australia<br>Werren and Trenerry, 1993 [81]	257 total	Lowland rainforest	Adult	Frequency	Formicidae (ants excl. bull ants and green tree ants)<br>74%	Beetles (excl. weevils)<br>68%	Orthoptera (crickets and katydids)<br>36%	% frequency from Table 1; Curculionidae (weevils) a close quarternary (31%) prey.
Introduced	West Pacific	American Samoa<br>Grant, 1996 [104]	26	Suburban	Subadult - adult	AII	Coleoptera (beetles)<br>37%	Lepidoptera (moths)<br>34%	Diplopoda (millipedes)<br>9%	% frequency and % number from Table 3 used to calculate AII; Dermaptera (earwigs) a close quarternary (8%) prey.
Introduced	West Pacific	American Samoa<br>Grant, 1996 [104]	28	Forest	Subadult - adult	AII	Diplopoda (millipedes)<br>58%	Lepidoptera (moths)<br>15%	Coleoptera (beetles)<br>12%	% frequency and % number from Table 3 used to calculate AII; Hymenoptera (ants?) a close quarternary (12%) prey.
Introduced	West Pacific	Rota, Northern Mariana Islands<br>Reed et al., 2007 [10]	284	Golf course and village	Juvenile - adult	Frequency	Coleoptera (beetles)<br>52%	Formicidae (ants)<br>39%	Diplopoda (millipedes)<br>33%	% frequency calculated from Table 1 using stomachs with identifiable prey; Orthoptera (grasshoppers) a close quarternary (31%) prey.
Introduced	West Pacific	Japan<br>Kidera et al., 2008 [83]	18	Pond	Subadult - adult	IRI	Hymenoptera (mostly ants)<br>36%	Lepidoptera (moths and butterflies)<br>26%	Coleoptera (beetles)<br>22%	% frequency, % number, and % volume from Table 1 used to calculate % IRI.
Introduced	West Pacific	Japan<br>Kidera et al., 2008 [83]	13	Forest	Juvenile - adult	IRI	Coleoptera (beetles)<br>35%	Hymenoptera (mostly ants)<br>34%	Hemiptera (true bugs)<br>15%	% frequency, % number, and % volume from Table 1 used to calculate % IRI.
Introduced	West Pacific	Japan<br>Kidera et al., 2008 [83]	27	Rice paddies	Subadult - adult	IRI	Hymenoptera (mostly ants)<br>55%	Diptera larvae (flies)<br>18%	Coleoptera (beetles)<br>10%	% frequency, % number, and % volume from Table 1 used to calculate % IRI.
Introduced	West Pacific	Queensland, Australia<br>Heise-Pavlov and Longway, 2011 [84]	81 total	Agroforestry plantation	Juvenile	Number	Hymenoptera (ants)<br>71%	Coleoptera (beetles)<br>22%	Unidentified Larvae<br>2%	% number calculated from the averaged numbers for taxa in Table 1.
Introduced	West Pacific	Queensland, Australia<br>Heise-Pavlov and Longway, 2011 [84]	81 total	Agroforestry trail	Juvenile	Number	Hymenoptera (ants)<br>84%	Coleoptera (beetles)<br>9%	Hemiptera (true bugs)<br>2%	% number calculated from the averaged numbers for taxa in Table 1.
Introduced	West Pacific	Phillipines<br>Pamintuan and Starr, 2016 [31]	58	Urban - wet season	n/a	Number	Formicidae (ants, including *Solenopsis* and *Oecophylla*)<br>79%	Scarabaeinae (dung beetles)<br>5%	Coleoptera (beetles)<br>4%	% number From Table 1; seasonal diff.
Introduced	West Pacific	Phillipines<br>Pamintuan and Starr, 2016 [31]	71	Urban - dry season	n/a	Number	Isoptera/Blattodea (termites)<br>76%	Formicidae (ants, including *Solenopsis* and *Oecophylla*)<br>9%	Tenebrionidae (darkling beetles)<br>8%	% number From Table 1; seasonal diff.
Introduced	West Pacific	Davao City, Phillipines<br>Apayor-Ynot et al., 2017 [105]	24	Park and subdivision	n/a	Frequency	Coleoptera (beetles)<br>75%	Blattodea (cockroaches)<br>71%	Isoptera/Blattodea (termites)<br>36%	% frequency from Table 1.
Introduced	West Pacific	Queensland, Australia<br>Lettoof et al., 2018 [106]	57 pellets	Bird rookery and road	Juvenile - adult	Number	Coleoptera (beetles)<br>43%	Hymenoptera (ants)<br>34%	Isoptera/Blattodea (termites)<br>13%	% number based on text in the results section.
Introduced	West Pacific	Philippines<br>Shuman-Goodier et al., 2019 [107]	101	Rice paddies	n/a	AII	*Solenopsis geminata* (tropical fire ant)<br>48%	*Solenopsis* sp. (fire ants)<br>35%	Formicidae (unidentified ants)<br>14%	% frequency and % number from Table 2 used to calculate % AII.
Introduced	West Pacific	New South Wales, Australia<br>Kaiser et al., 2022 [32]	195	Burned/unburned forest	Adult female	Number	Hymenoptera (ants)<br>50%	Coleoptera (beetles)<br>35%	Myriapods (primarily millipedes)<br>6%	% number calculated by sex from a supplementary spreadsheet; diff. between sexes.
Introduced	West Pacific	New South Wales, Australia<br>Kaiser et al., 2022 [32]	291	Burned/unburned forest	Adult male	Number	Hymenoptera (ants)<br>61%	Coleoptera (beetles)<br>28%	Lepidoptera (moths and butterflies)<br>6%	% number calculated by sex from a supplementary spreadsheet; diff. between sexes.

References

8. Zug, G.R.; Zug, P.B. The marine toad, *Bufo marinus*: A natural history resume of native populations. *Smithson. Contrib. Zool.* **1979**, *284*, 1–58. https://doi.org/10.5479/si.00810282.284.
9. Parmalee, J.R. Trophic ecology of a tropical anuran assemblage. Scientific Papers. *Nat. Hist. Mus. Univ. Kans.* **1999**, *11*, 1–59.
10. Reed, R.N.; Bakkegard, K.A.; Desy, G.E.; Plentovich, S.M. Diet composition of the invasive cane toad (*Chaunus marinus*) on Rota, Northern Mariana Islands. *Pac. Conserv. Biol.* **2007**, *13*, 219–222. https://doi.org/10.1071/PC070219.
16. Krakauer, T. The ecology of the neotropical toad, *Bufo marinus*, in south Florida. *Herpetologica* **1968**, *24*, 214–221.
20. Rossi, J.V. Bufo Marinus in Florida: Some Natural History and Its Impact on Native Vertebrates. Master's Thesis, University of South Florida, Tampa, FL, USA, December 1981. Available online: https://usf-flvc.primo.exlibrisgroup.com/discovery/fulldisplay?context=L&vid=01FALSC_USF:USF&search_scope=MyInst_and_CI&tab=Everything&docid=alma99379536742206599.
26. Dexter, R.R. The food habits of the imported toad *Bufo marinus* in the sugar cane sections of Porto Rico. *Bull. Fourth Congr. Int. Soc. Sugar Cane Technol.* **1932**, *74*, 1–6.
27. Wolcott, G.N. What the giant Surinam toad, *Bufo marinus* L., is eating now in Puerto Rico. *J. Agric. Univ. Puerto Rico* **1937**, *21*, 79–84. https://doi.org/10.46429/jaupr.v21i1.14345.
28. Zug, G.R.; Lindgren, E.; Pippet, J.R. Distribution and ecology of the marine toad, *Bufo marinus*, in Papua New Guinea. *Pac. Sci.* **1975**, *29*, 31–50.
29. Evans, M.; Lampo, M. Diet of *Bufo marinus* in Venezuela. *J. Herpetol.* **1996**, *30*, 73–76. https://doi.org/10.2307/1564710.
30. Linzey, D.W.; Deel, A.C.; Adams, S.J.; Linzey, J.B. Spring and summer foods of *Bufo marinus* (Amphibia: Bufonidae) and *Eleutherodactylus johnstonei* (Amphibia: Leptodactylidae) in Bermuda. *J. Elisha Mitchell Sci. Soc.* **1998**, *114*, 125–136.
31. Pamintuan, P.E.; Starr, C.K. Diet of the giant toad, *Bufo marinus* (Amphibia: Salientia), in a coastal habitat of the Philippines. *Trop. Agric.* **2016**, *93*, 323–327.
32. Kaiser, S.W.; Greenlees, M.J.; Shine, R. Sex-based differences in the use of post-fire habitats by invasive cane toads (*Rhinella marina*). *Sci. Rep.* **2022**, *12*, 10610. https://doi.org/10.1038/s41598-022-14697-7.
33. Meshaka, W.E., Jr.; Powell, R. Diets of the native southern toad (*Anaxyrus terrestris*) and the exotic cane toad (*Rhinella marina*) form a single site in south-central Florida. *Fla. Sci.* **2010**, *73*, 175–179.
49. Smoak, C.H.; Schmid, J.R. Feeding habits of the non-native Mayan cichlid, *Mayaheros uropthalmus*, in estuarine tributaries of southwest Florida. *Gulf Caribb. Res.* **2021**, *32*, 57–66. https://doi.org/10.18785/gcr.3201.08.
51. Pinkas, L.; Oliphant, M.S.; Iverson, I.L.K. Food habits of albacore, Bluefin tuna, and bonito in California waters. *Calif. Dep. Fish Game Fish Bull.* **1971**, *152*, 1–105.
81. Werren, G.L.; Trenerry, M.P. Size and diet of *Bufo marinus* in rainforest of northeastern Queensland. *Mem. Qld. Mus.* **1993**, *34*, 240.
82. Strüssmann, C.; Ribeiro do Vale, M.B.; Meneghini, M.H.; Magnusson, W.E. Diet and foraging mode of *Bufo marinus* and *Leptodactylus ocellatus*. *J. Herpetol.* **1984**, *18*, 138–146. https://doi.org/10.2307/1563741.
83. Kidera, N.; Tandavanitj, N.; Oh, D.; Nakanishi, N.; Satoh, A.; Denda, T.; Izawa, M.; Ota, H. Dietary habits of the introduced cane toad *Bufo marinus* (Amphibia: Bufonidae) on Ishigakijima, Southern Ryukyus, Japan. *Pac. Sci.* **2008**, *62*, 423–430. https://doi.org/10.2984/1534-6188(2008)62[423:DHOTIC]2.0.CO;2.
84. Heise-Pavlov, S.R.; Longway, L.J. Diet and dietary selectivity of cane toads (*Rhinella marina*) in restoration sites: A case study in Far North Queensland, Australia. *Ecol. Manag. Restor.* **2011**, *12*, 230–232. https://doi.org/10.1111/j.1442-8903.2011.00603.x.
87. Sampedro-Marín, A.C.; Angulo Villalba, Y.Y.; Arrieta Díaz, F.I.; Domínguez Atencia, D.M. Alimentación de *Bufo marinus* (Linnaeus, 1758) (Bufonidae: Anura), en una localidad de Sucre, Colombia. *Caldasia* **2011**, *33*, 495–505.
96. Noble, G.K. The amphibians collected by the American Museum expedition to Nicaragua in 1916. *Bull. Am. Mus. Nat. Hist.* **1918**, *38*, 311–347.
97. Weber, N.H. The food of the giant toad, *Bufo marinus* (L.), in Trinidad and British Guiana with special reference to the ants. *Ann. Entomol. Soc. Am.* **1938**, *31*, 498–503. https://doi.org/10.1093/aesa/31.4.499.
98. Duellman, W.E. The biology of an equatorial herpetofauna in Amazonian Ecuador. *Misc. Publ. Univ. Kans. Mus. Nat. Hist.* **1978**, *65*, 1–352.
99. Cabrera, J.; Barrantes, R.; Rodríguez, D. Hábitos alimentarios de *Bufo marinus* (Anura Bufonidae) en Costa Rica. *Rev. De Biol. Trop.* **1996**, *44*, 702–703.
100. Isaacs, P.; Hoyos, J.M. Diet of the cane toad in different vegetation covers in the productive systems of the Colombian coffee region. *South Am. J. Herpetol.* **2010**, *5*, 45–50. https://doi.org/10.2994/057.005.0105.
101. Blanco-Torres, A.; Duré, M.I.; Bonilla, M.A.; Cagnolo, L. Predator-prey interactions in anurans of the tropical dry forests of the Colombian Caribbean: A functional approach. *Biotropica* **2020**, *52*, 730–737. https://doi.org/10.1111/btp.12779.
102. Hinckley, A.D. Diet of the giant toad, *Bufo marinus* (L.), in Fiji. *Herpetologica* **1963**, *18*, 253–259.
103. Bailey, P. Food of the marine toad, *Bufo marinus*, and six species of skink in a cacao plantation in New Britain, Papua New Guinea. *Aust. Wildl. Res.* **1976**, *3*, 185–188. https://doi.org/10.1071/WR9760185.
104. Grant, G.S. Prey of the introduced *Bufo marinus* on American Samoa. *Herpetol. Rev.* **1996**, *27*, 67–69.
105. Apayor-Ynot, C.L.; Tan, S.N.; Lim, N.K.; Delima-Baron, E.M.; Mohagan, A.B. Diet of cane toads (*Rhinella marina*) collected from areas adjacent to human dwellings in Davao City, Philippines. *Imp. J. Interdiscip. Res.* **2017**, *3*, 640–642.
106. Lettoof, D.C.; Lyons, J.A.; Shine, R.; Maniel, G.; Mayer, M.; Natusch, D.J.D. Cane toads beneath bird rookeries: Utilization of a natural disturbance by an invasive species. *Curr. Zool.* **2018**, *64*, 433–439. https://doi.org/10.1093/cz/zox041.
107. Shuman-Goodier, M.E.; Diaz, M.I.; Almazan, M.L.; Singleton, G.R.; Hadi, B.A.R.; Propper, C.R. Ecosystem hero and villain: Native frog consumes rice pests, while the invasive cane toad feasts on beneficial arthropods. *Agric. Ecosyst. Environ.* **2019**, *279*, 100–108. https://doi.org/10.1016/j.agee.2019.04.008.
