# Supplementary material for: Faunal Diet of Adult Cane Toads, Rhinella marina, in the Urban Landscape of Southwest Florida
Source: Animals (Basel). 2023 Sep 13;13(18):2898. doi: 10.3390/ani13182898 (PMC10526063; doi:10.3390/ani13182898)
Supplement: Supplementary file 1 [file animals-13-02898-s001.zip › Table S2. Cane toad prey taxa list.pdf]

Table S2. Cumulative list of the number (N), volume (V), frequency (F), and index of relative importance (IRI) of prey taxa for cane toads collected in southwest Florida golf course communities. Taxa with the highest IRI are in bold.

Prey taxonomy (Common name)	N	% N	V	% V	F	% F	%IRI
Annelida (Segmented worms)	-	-	-	-	-	-	-
Clitellata (Collar worms)	-	-	-	-	-	-	-
Opisthopora (Terrestrial worms)	18	0.13	10.50	0.96	11	4.60	0.12
Lumbricidae (Earthworms)	38	0.27	2.50	0.23	3	1.26	0.02
Platyhelminthes (Flatworms)	-	-	-	-	-	-	-
Trematoda (Parasitic worms)	6	0.04	0.02	<0.01	2	0.84	<0.01
Arthropoda (Invertebrate animals such as crabs, spiders, and insects)	3	0.02	<0.01	<0.01	1	0.42	<0.01
Chilopoda (Centipedes)	11	0.08	0.01	<0.01	5	2.09	<0.01
Geophilomorpha (Soil centipedes)	2	0.01	0.11	0.01	2	0.84	<0.01
Collembola (Springtails)	-	-	-	-	-	-	-
Poduromorpha	2	0.01	0.10	0.01	1	0.42	<0.01
Diplopoda (Millipedes)	18	0.13	2.40	0.22	5	2.09	0.02
Julida (Julid millipedes)	-	-	-	-	-	-	-
Parajulidae (Cylindrical millipedes)	43	0.31	9.71	0.89	17	7.11	0.20
Polydesmida (Flat-backed millipedes)	13	0.09	0.90	0.08	1	0.42	<0.01
Paradoxosomatidae (Dragon millipedes)	49	0.35	4.01	0.37	13	5.44	0.09
Asiomorpha coarctata (Long-flange millipedes)	186	1.33	20.50	1.87	34	14.23	1.10
Oxidus gracilis (Greenhouse millipedes)	14	0.10	1.55	0.14	2	0.84	<0.01
Polyzoniida (Polyzoniid millipedes)	-	-	-	-	-	-	-
Siphonotidae	-	-	-	-	-	-	-
Rhinotus purpureus (Purple womble millipede)	6	0.04	0.01	<0.01	1	0.42	<0.01
Spirobolida (Round-backed millipedes)	6	0.04	4.50	0.41	3	1.26	0.01
Rhinocricidae	-	-	-	-	-	-	-
***Anadenobolus monilicornis* (Yellow-banded millipedes)**	**631**	**4.52**	**298.47**	**27.29**	**89**	**37.24**	**28.48**
Euchelicerata (Spiders, mites, scorpions, and related taxa)	1	0.01	<0.01	<0.01	1	0.42	<0.01
Araneae (Spiders)	20	0.14	1.62	0.15	14	5.86	0.04
Araneidae (Orbweaver spiders)	-	-	-	-	-	-	-
Gasteracantha cancriformis (Spinybacked orbweavers)	1	0.01	0.04	<0.01	1	0.42	<0.01
Clubionidae (Sac spiders)	-	-	-	-	-	-	-
Clubiona (Leafcurling sac spiders)	5	0.04	0.80	0.07	3	1.26	<0.01
Lycosidae (Wolf spiders)	9	0.06	1.79	0.16	8	3.35	0.02
Allocosa (Wolf spiders)	1	0.01	0.01	<0.01	1	0.42	<0.01
Tigrosa ("Fierce like a tiger" wolf spiders)	1	0.01	0.50	0.05	1	0.42	<0.01
Tigrosa annexa (Wolf spiders)	12	0.09	5.75	0.53	11	4.60	0.07
Trebacosa marxi (Dull wolf spiders)	1	0.01	0.20	0.02	1	0.42	<0.01
Pisauridae (Nursery web spiders)	1	0.01	0.40	0.04	1	0.42	<0.01
Dolomedes triton (Six-spotted fishing spiders)	13	0.09	7.80	0.71	9	3.77	0.07
Tetragnatha (Long-jawed orb weavers)	2	0.01	0.41	0.04	2	0.84	<0.01
Mesostigmata (Predatory mites)	3	0.02	<0.01	<0.01	2	0.84	<0.01
Sarcoptiformes (Mites)	15	0.11	0.41	0.04	3	1.26	<0.01
Galumnidae (Mites and ticks)	1	0.01	<0.01	<0.01	1	0.42	<0.01
Trombidiformes (Mites)	1	0.01	<0.01	<0.01	1	0.42	<0.01
Insecta (Beetles, ants, earwigs, butterflies, and related taxa)	121	0.87	5.21	0.48	29	12.13	0.39
Blattodea (Cockroaches and termites)	5	0.04	0.41	0.04	2	0.84	<0.01
Blaberidae (Giant cockroaches)	1	0.01	<0.01	<0.01	1	0.42	<0.01
Pycnoscelus	1	0.01	0.80	0.07	1	0.42	<0.01
Pycnoscelus surinamensis (Surinam cockroaches)	12	0.09	7.95	0.73	5	2.09	0.04
Coleoptera (Beetles)	375	2.69	20.43	1.87	65	27.20	2.98
Carabidae (Ground beetles)	9	0.06	1.50	0.14	4	1.67	0.01
Agonum	2	0.01	0.20	0.02	2	0.84	<0.01
Harpalus	1	0.01	0.40	0.04	1	0.42	<0.01
Loxandrus	1	0.01	0.20	0.02	1	0.42	<0.01
Omophron labiatum (Large-lipped round sand beetles)	1	0.01	0.01	<0.01	1	0.42	<0.01
Pasimachus	3	0.02	1.32	0.12	3	1.26	<0.01
Scarites	3	0.02	1.60	0.15	3	1.26	0.01
Selenophorus	1	0.01	0.10	0.01	1	0.42	<0.01
Semiardistomis	6	0.04	0.50	0.05	3	1.26	<0.01
Chrysomelidae (Leaf beetles)	-	-	-	-	-	-	-
Caryobruchus gleditsiae (Palm seed weevils)	1	0.01	0.40	0.04	1	0.42	<0.01
Donacia (Aquatic leaf beetles)	2	0.01	0.40	0.04	2	0.84	<0.01
Coccinellidae (Lady bugs)	3	0.02	<0.01	<0.01	2	0.84	<0.01
Harmonia axyridis (Multicolored Asian ladybugs)	1	0.01	0.05	<0.01	1	0.42	<0.01
Curculionidae (True weevils)	112	0.80	10.65	0.97	11	4.60	0.20
Coccotrypes distinctus (Bark beetles)	1	0.01	<0.01	<0.01	1	0.42	<0.01
Myllocerus undecimpustulatus undatus (Sri Lankan weevils)	1	0.01	0.10	0.01	1	0.42	<0.01
Sphenophorus (Billbugs)	232	1.66	24.52	2.24	16	6.69	0.63
Sphenophorus inaequalis (Uneven billbugs)	259	1.86	26.37	2.41	29	12.13	1.24
***Sphenophorus venatus* (Hunting billbugs)**	**1556**	**11.15**	**128.16**	**11.72**	**86**	**35.98**	**19.78**
Xyleborus (Ambrosia beetles)	1	0.01	<0.01	<0.01	1	0.42	<0.01
Dryopidae (Long-toed water beetles)	-	-	-	-	-	-	-
Pelonomus	1	0.01	0.03	<0.01	1	0.42	<0.01
Dytiscidae (Predaceous diving beetles)	1	0.01	0.10	0.01	1	0.42	<0.01
Elateridae (Click beetles)	54	0.39	3.72	0.34	18	7.53	0.13
Conoderus ('Cone-necked')	2	0.01	0.05	<0.01	1	0.42	<0.01
Conoderus fallii (Southern potato wireworms)	69	0.49	4.34	0.40	38	15.90	0.34
Conoderus pictus (Click beetles)	1	0.01	0.01	<0.01	1	0.42	<0.01
Heteroderes amplicollis (Gulf wireworms)	8	0.06	0.11	0.01	4	1.67	<0.01
Rismethus	11	0.08	0.22	0.02	8	3.35	0.01
Heteroceridae (Variegated mud-loving beetles)	-	-	-	-	-	-	-
Tropicus pusillus (Variegated mud-loving beetles)	1	0.01	0.50	0.05	1	0.42	<0.01
Hybosoridae (Scavenger scarab beetles)	-	-	-	-	-	-	-
Hybosorus illigeri (Scavenger scarab beetles)	97	0.69	15.52	1.42	22	9.21	0.47
Hydrophilidae (Water scavanger beetles)	1	0.01	0.10	0.01	1	0.42	<0.01
Tropisternus	3	0.02	0.20	0.02	1	0.42	<0.01
Tropisternus natator (Water scavanger beetles)	3	0.02	0.71	0.06	3	1.26	<0.01
Nitidulidae (Sap beetles)	-	-	-	-	-	-	-
Stelidota (Sap-feeding beetles)	1	0.01	<0.01	<0.01	1	0.42	<0.01
Ptilodactylidae (Toe-winged beetles)	2	0.01	0.10	0.01	2	0.84	<0.01
Ptilodactyla (Toe-winged beetles)	26	0.19	0.70	0.06	11	4.60	0.03
Scarabaeidae (Scarab beetles)	131	0.94	25.24	2.31	25	10.46	0.82
Anomala	8	0.06	1.61	0.15	4	1.67	0.01
Anomala innuba (Shining leaf chafers)	3	0.02	0.76	0.07	2	0.84	<0.01
Anomala marginata (Shining leaf chafers)	10	0.07	5.60	0.51	7	2.93	0.04
Ataenius (Dung beetles)	15	0.11	0.21	0.02	4	1.67	0.01
Ataenius imbricatus (Aphodiine dung beetles)	2	0.01	0.05	<0.01	1	0.42	<0.01
Cyclocephala (Masked chafers)	23	0.16	7.24	0.66	10	4.18	0.08
Dyscinetus (Rice beetles)	1	0.01	1.00	0.09	1	0.42	<0.01
Dyscinetus morator (Rice beetles)	7	0.05	5.60	0.51	6	2.51	0.03
Euphoria sepulcralis (Dark flower scarabs)	48	0.34	5.57	0.51	14	5.86	0.12
Geopsammodius (Aphodiine dung beetles)	7	0.05	0.30	0.03	2	0.84	<0.01
Onthophagus hecate blatchleyi (Scooped scarabs)	1	0.01	0.10	0.01	1	0.42	<0.01
Phyllophaga bruneri (Cuban May beetles)	77	0.55	31.03	2.84	26	10.88	0.89
Tomarus (Carrot beetles)	13	0.09	12.50	1.14	9	3.77	0.11
Staphylinidae (Rove beetles)	2	0.01	0.18	0.02	2	0.84	<0.01
Tenebrionidae (Darkling beetles)	-	-	-	-	-	-	-
Gondwanocrypticus	1	0.01	<0.01	<0.01	1	0.42	<0.01
Platydema	51	0.37	5.34	0.49	8	3.35	0.07
Dermaptera (Earwigs)	21	0.15	0.64	0.06	8	3.35	0.02
Carcinophoridae (Earwigs)	2	0.01	0.02	<0.01	1	0.42	<0.01
Euborellia	1	0.01	0.05	<0.01	1	0.42	<0.01
Euborellia annulipes (Ring-legged earwigs)	20	0.14	1.20	0.11	13	5.44	0.03
Labiduridae (Striped earwigs)	8	0.06	1.12	0.10	6	2.51	0.01
Labidura	1	0.01	<0.01	<0.01	1	0.42	<0.01
Labidura riparia (Shore earwigs)	319	2.28	48.13	4.40	62	25.94	4.17
Diptera (Flies)	75	0.54	5.93	0.54	6	2.51	0.07
Calliphoridae (Carrion flies)	5	0.04	0.70	0.06	3	1.26	<0.01
Cecidomyiidae (Gall midges)	1	0.01	<0.01	<0.01	1	0.42	<0.01
Chloropidae (Chloropid flies)	3	0.02	<0.01	<0.01	2	0.84	<0.01
Sciaridae (Dark-winged fungus gnats)	1	0.01	<0.01	<0.01	1	0.42	<0.01
Stratiomyidae (Solider fly maggots)	1	0.01	0.05	<0.01	1	0.42	<0.01
Hemiptera (True bugs, hoppers, aphids, and allies)	5	0.04	0.40	0.04	3	1.26	<0.01
Belostomatidae (Giant water bugs)	-	-	-	-	-	-	-
Belostoma (Giant water bugs)	1	0.01	0.30	0.03	1	0.42	<0.01
Lethocerus (*Benacus*) *griseus* (Eastern toe biters)	1	0.01	7.50	0.69	1	0.42	0.01
Blissidae	-	-	-	-	-	-	-
Blissus (Chinch bugs)	1	0.01	<0.01	<0.01	1	0.42	<0.01
Cicadidae (Cicadas)	1	0.01	3.50	0.32	1	0.42	<0.01
Coreidae (Leaf-footed bugs)	1	0.01	0.30	0.03	1	0.42	<0.01
Cydnidae (Burrowing bugs)	1	0.01	0.01	<0.01	1	0.42	<0.01
Ochteridae (Velvety shore bugs)	-	-	-	-	-	-	-
Ochterus (Velvety shore bugs)	1	0.01	<0.01	<0.01	1	0.42	<0.01
Tropiduchidae (Planthoppers)	-	-	-	-	-	-	-
Pelitropis rotulata (tropiduchid planthopper)	1	0.01	0.10	0.01	1	0.42	<0.01
Hymenoptera (Ants, bees, wasps, and sawflies)	23	0.16	2.41	0.22	12	5.02	0.05
Apidae (Honey bees, bumble bees, and allies)	3	0.02	0.50	0.05	3	1.26	<0.01
Apis (Honey bees)	1	0.01	0.50	0.05	1	0.42	<0.01
Apis mellifera (Western honey bees)	145	1.04	25.60	2.34	7	2.93	0.24
Crabronidae (Crabronid wasps)	-	-	-	-	-	-	-
Larra bicolor (Mole cricket hunters)	1	0.01	0.50	0.05	1	0.42	<0.01
Formicidae (Ants)	249	1.78	2.65	0.24	27	11.30	0.55
Brachymyrmex (Rover ants)	1317	9.43	3.26	0.30	10	4.18	0.98
Brachymyrmex obscurior (Obscure rover ants)	545	3.90	1.94	0.18	6	2.51	0.25
Camponotus (Carpenter ants)	2	0.01	<0.01	<0.01	2	0.84	<0.01
Camponotus floridanus (Florida carpenter ants)	472	3.38	10.84	0.99	32	13.39	1.41
Camponotus sexguttatus (Six-spotted carpenter ants)	1	0.01	0.01	<0.01	1	0.42	<0.01
Camponotus tortuganus (Tortugas carpenter ants)	1	0.01	<0.01	<0.01	1	0.42	<0.01
Crematogaster (Acrobat ants)	2	0.01	0.05	<0.01	2	0.84	<0.01
Hypoponera opaciceps (Granulate mini-ponerine ants)	1	0.01	<0.01	<0.01	1	0.42	<0.01
Hypoponera pacior (Mini-ponerine ants)	1	0.01	<0.01	<0.01	1	0.42	<0.01
Nylanderia bourbonica (Robust Crazy ants)	2	0.01	0.15	0.01	2	0.84	<0.01
Odontomachus (Giant trap-jaw ants)	55	0.39	2.63	0.24	28	11.72	0.18
Odontomachus brunneus (Florida trap-jaw ants)	27	0.19	1.60	0.15	3	1.26	0.01
Odontomachus ruginodis (Rough-node Trap-jaw ants)	13	0.09	<0.01	<0.01	1	0.42	<0.01
***Pheidole* (Big-headed ants)**	**4246**	**30.41**	**18.39**	**1.68**	**81**	**33.89**	**26.15**
Pheidole bilimeki (Big-headed ants)	165	1.18	1.68	0.15	3	1.26	0.04
Pheidole dentigula (Woodland big-headed ants)	651	4.66	2.05	0.19	15	6.28	0.73
Pheidole flavens (Yellow big-headed ants)	324	2.32	0.41	0.04	4	1.67	0.09
Platythyrea punctata (Common silvery hunter ants)	2	0.01	0.01	<0.01	1	0.42	<0.01
Pseudomyrmex gracilis (Big bicolored slender twig ants)	3	0.02	0.02	<0.01	3	1.26	<0.01
Solenopsis invicta (Red imported fire ants)	1	0.01	0.01	<0.01	1	0.42	<0.01
Tetramonium bicarinatum (Bicolored pennant ants)	2	0.01	<0.01	<0.01	1	0.42	<0.01
Wasmannia (common name unknown)	2	0.01	<0.01	<0.01	1	0.42	<0.01
Wasmannia auropunctata (Little fire ants)	31	0.22	0.10	0.01	3	1.26	0.01
Ichneumonidae (Parasitoid wasps)	-	-	-	-	-	-	-
Ophion (Short-tailed ichneumon wasps)	1	0.01	0.50	0.05	1	0.42	<0.01
Pompilidae (Spider wasps)	1	0.01	0.30	0.03	1	0.42	<0.01
Lepidoptera (Butterflies/moths)	490	3.51	55.20	5.05	63	26.36	5.42
Psychidae (Bagworm moths)	3	0.02	0.32	0.03	3	1.26	<0.01
Odonata (Dragonflies and damselflies)	-	-	-	-	-	-	-
Libellulidae (Skimmer dragonflies)	1	0.01	0.70	0.06	1	0.42	<0.01
Orthoptera (Grasshoppers, crickets, and katydids)	1	0.01	<0.01	<0.01	1	0.42	<0.01
Gryllotalpidae (Mole crickets)	15	0.11	13.80	1.26	14	5.86	0.19
Neoscapteriscus borellii (Southern mole crickets)	18	0.13	17.30	1.58	15	6.28	0.26
Zygentoma (Silverfish, fishmoths, and firebrats)	-	-	-	-	-	-	-
Lepismatidae (Silverfish)	1	0.01	<0.01	<0.01	1	0.42	<0.01
Malacostraca (Crustaceans)	-	-	-	-	-	-	-
Decapoda (Crabs, lobsters, crayfish, and shrimp)	-	-	-	-	-	-	-
Cambaridae (Crayfish)	1	0.01	2.50	0.23	1	0.42	<0.01
Isopoda (Woodlice)	11	0.08	2.40	0.22	6	2.51	0.02
Mollusca (Invertebrate animals such as clams, snails, slugs, and squid)	-	-	-	-	-	-	-
Gastropoda (Snails and slugs)	17	0.12	1.15	0.10	8	3.35	0.02
Basommatophora (Snails)	-	-	-	-	-	-	-
Planorbidae (Ramshorn snails)	1	0.01	1.10	0.10	1	0.42	<0.01
Micromenetus floridensis (Penny sprite)	3	0.02	1.65	0.15	3	1.26	0.01
Bivalvia (molluscs such as clams, oysters, cockles, mussels, scallops)	5	0.04	0.71	0.07	4	1.67	<0.01
Stylommatophora (Common land snails and slugs)	2	0.01	0.20	0.02	2	0.84	<0.01
Polygyridae (Polygyrid snails)	1	0.01	0.10	0.01	1	0.42	<0.01
Polygyra cereolus (Air-breathing land snails)	42	0.30	2.84	0.26	15	6.28	0.08
Pupillidae (Chrysalis snails)	2	0.01	0.03	<0.01	2	0.84	<0.01
Pupoides (Dagger snails)	19	0.14	1.40	0.13	13	5.44	0.03
Chordata (Vertebrate animals)	6	0.04	2.00	0.18	3	1.26	0.01
Amphibia (Amphibians)	2	0.01	2.60	0.24	2	0.84	0.01
Anura (Frogs and toads)	1	0.01	2.00	0.18	1	0.42	<0.01
Bufonidae (True toads)	8	0.06	20.90	1.91	4	1.67	0.08
Hylidae (Tree frogs and allies)	4	0.03	15.80	1.44	3	1.26	0.04
Osteopilus septentrionalis (Cuban treefrogs)	1	0.01	18.10	1.65	1	0.42	0.02
Aves (Birds)	1	0.01	0.90	0.08	1	0.42	<0.01
Anseriformes (Waterfowl)	-	-	-	-	-	-	-
Anatidae (Ducks, geese, and swans)	4	0.03	3.80	0.35	4	1.67	0.02
Columbiformes (Pigeons, doves, dodoes, and solitaires)	-	-	-	-	-	-	-
Columbidae (Pigeons and doves)	1	0.01	0.80	0.07	1	0.42	<0.01
Zenaida macroura (Mourning doves)	1	0.01	3.25	0.30	1	0.42	<0.01
Reptilia (Reptiles)	-	-	-	-	-	-	-
Squamata (Scaled reptiles)	-	-	-	-	-	-	-
Dactyloidae (Anoles)	1	0.01	1.90	0.17	1	0.42	<0.01
Anolis (Western Antillean anoles)	2	0.01	2.25	0.21	2	0.84	<0.01
Anolis sagrei (Brown anoles)	1	0.01	6.10	0.56	1	0.42	0.01
Typhlopidae (Blind snakes)	-	-	-	-	-	-	-
Indotyphlops braminus (Brahminy blindsnakes)	3	0.02	4.20	0.38	3	1.26	0.01
Testudines (Turtles)	-	-	-	-	-	-	-
Trionychidae (Softshell turtles)	-	-	-	-	-	-	-
Apalone ferox (Florida softshell turtles)	1	0.01	7.50	0.69	1	0.42	0.01
Teleostei (Fish)	1	0.01	<0.01	<0.01	1	0.42	<0.01
Mammalia (Mammals)	3	0.02	13.11	1.20	3	1.26	0.04
Rodentia (Rodents)	1	0.01	6.10	0.56	1	0.42	0.01
